# Influence of Flame Retardants on the Melt Dripping Behaviour of Thermoplastic Polymers

**DOI:** 10.3390/ma8095267

**Published:** 2015-08-27

**Authors:** Melissa Matzen, Baljinder Kandola, Christian Huth, Bernhard Schartel

**Affiliations:** 1 BAM Federal Institute for Materials Research and Testing, Unter den Eichen 87, Berlin 12205, Germany; E-Mails: melissa.matzen@bam.de (M.M.); christian.huth@bam.de (C.H.);; 2 Institute for Materials Research and Innovation, University of Bolton, Deane Road, Bolton BL3 5AB, UK; E-Mail: B.Kandola@bolton.ac.uk

**Keywords:** fire retardant, viscosity, melt dripping, reaction-to-small-flame, UL 94

## Abstract

Melt flow and dripping of the pyrolysing polymer melt can be both a benefit and a detriment during a fire. In several small-scale fire tests addressing the ignition of a defined specimen with a small ignition source, well-adjusted melt flow and dripping are usually beneficial to pass the test. The presence of flame retardants often changes the melt viscosity crucially. The influence of certain flame retardants on the dripping behaviour of four commercial polymers, poly(butylene terephthalate) (PBT), polypropylene (PP), polypropylene modified with ethylene-propylene rubber (PP-EP) and polyamide 6 (PA 6), is analysed based on an experimental monitoring of the mass loss due to melt dripping, drop size and drop temperature as a function of the furnace temperature applied to a rod-shaped specimen. Investigating the thermal transition (DSC), thermal and thermo-oxidative decomposition, as well as the viscosity of the polymer and collected drops completes the investigation. Different mechanisms of the flame retardants are associated with their influence on the dripping behaviour in the UL 94 test. Reduction in decomposition temperature and changed viscosity play a major role. A flow limit in flame-retarded PBT, enhanced decomposition of flame-retarded PP and PP-EP and the promotion of dripping in PA 6 are the salient features discussed.

## 1. Introduction

Fire behaviour is reflected by the response of defined specimens or components in distinct fire scenarios. The fire properties observed are not material properties, but are based on complex interactions of different phenomena in the gas phase and the condensed phase. The pyrolysis of the polymer in the condensed phase, the oxidation of volatile fuel in the gas phase (flame), the mass transport to the flame and the heat transport to the pyrolysis front are the main phenomena controlling burning. Each of these main actors is merely a category comprised of a dozen sub-phenomena. For instance, the pyrolysis includes the processes of heating the polymer, melting, decomposition, charring, and so on. Furthermore, the different processes interact with each other. The function of flame retardants usually involves several phenomena, both chemical and physical, at the same time. Apart from thermal insulation through intumescence, the role of flame retardants in changing the physical phenomena is often underestimated or neglected. This may be misleading in several systems, because the physical phenomena can be minor, but crucial or even become the main flame retardancy mode of action. For instance, heat absorption and reflection at the polymer surface, as well as heat shielding by hot surfaces are usually neglected, but the key role of changes in heat absorption on ignition in polymer/carbon nanoparticle nanocomposites has been presented [1,2]. An effective heat reflection (=infrared mirror) increases the time to ignition by an order of magnitude and, thus, reduces the maximum average rate of heat emission (MARHE) below 90 kW/m^2^ in the cone calorimeter at 50 kW/m^2^ irradiation [3]. Heat shielding (re-radiation of the hot surface) is the main general flame retardant mode of action for nanocomposites based on polymers that exhibit little or no charring [4,5]. In this paper, an important physical phenomenon in flame retardancy is addressed: the influence of flame retardants on dripping phenomena *versus* classification in the UL 94 test. Dripping in the UL 94 setup is controlled by thermophysical properties, such as the viscosity of the melt. This study elucidates the quite different impact of certain flame retardants on the dripping during reaction to small flame tests. It indicates how the change in properties is used to achieve a desired classification in commercial products.

Of course, melt flow and dripping of the pyrolysing polymer melt can be both a benefit and a detriment during a fire. Melt flow and dripping can reduce flame spread and even result in extinction, as it removes mass and heat from the actual pyrolysis zone. On the other hand, melt flow and dripping often provide an additional ignition source, an additional process of flame spread and even harbour the potential to start a pool fire separate from the original burning item. In several small-scale fire tests addressing the sustained ignition of a small specimen as a reaction to an ignition source at the beginning of a fire (oxygen index, UL 94 burning chamber, FMVSS 302, all kinds of Bunsen burner type tests, glow wire test), a well-adjusted melt flow and dripping are crucial to passing the test.

The vertical UL 94 test [6] is such a small-scale laboratory fire test used to classify the flammability of polymers under controlled laboratory conditions, and its protocol explicitly involves melt dripping. The main classification criterion in the UL 94 test is the extinction within a short time period of the defined specimen after removing the Bunsen burner-type ignition source. Furthermore, the ignition of a cotton pad below the specimen by flaming drops may be observed. Classifications V-0 and V-1 are obtained by immediate non-dripping self-extinction or non-flaming dripping self-extinction, roughly sketched, within 10 and 30 s, respectively. Achieving a V-0 classification is one of the most important goals for the flame retardancy of polymers. Immediate self-extinction based on flaming drops corresponds to the UL 94 classification V-2, which is still a very interesting classification for some products, such as films or thin-wall building products.

Recently, the quantification of dripping behaviour during burning has been the subject of various reports [7,8,9,10,11,12,13,14,15,16,17]. Most studies have been performed under fire operating conditions that resemble the UL 94 test [7,8,9,10,14,15]. The effect of convective heat on non-flaming melt dripping has been less intensively studied [12]. In the work reported in [12], six commercially available polymers without flame retardants were subjected to convective heat in a tube furnace, and their melt dripping behaviour was studied by measuring the mass loss as a function of time. Number, mass, shape, sizes and temperatures of individual drops were recorded, which varied depending on the polymer type and the furnace temperature [12,13]. From rheological and thermogravimetric analyses of the polymer and the fallen drops, it was established that during melt dripping, partial decomposition of the polymer occurs, the degree of which depends on the furnace temperature. Tests equivalent to UL 94 were also performed. It was demonstrated that dripping during fire tests is a complex interaction of physical and chemical processes, e.g., softening temperatures or viscosity changes due to chemical decomposition.

Adding flame retardants often changes the viscosity of the polymer melt, since they function as plasticizers or as reinforcing fillers. Further, the efficiency of many flame retardants depends on the viscosity of the modified polymers. In fact, flame retardants are often applied together with distinct adjuvants to adjust the dripping behaviour. Two different approaches are successfully realized in commercial systems: first, reducing viscosity to enhance dripping and, thus, achieving V-0 dripping or V-2 classification; second, inducing a flow limit (solid-like behaviour at low shear stresses below a yield point) to avoid dripping and, thus, achieving V-0 non-dripping. The latter case has been examined in detail for polycarbonate/acrylonitrile-butadiene-styrene (PC/ABS)/bisphenol A bis(diphenyl phosphate) (BDP)/polytetrafluoroethylene (PTFE) [10,18]. The flame retardant BDP functions as an efficient plasticizer for PC/ABS, so that not only PC/ABS, but also PC/ABS/BDP achieve V-2 classification. To get the desired non-dripping V-0 classification, the anti-dripping adjuvant PTFE was added, just as it is for several commercial flame-retardant thermoplastics. All kinds of anisotropic nanoparticles, such as layered silicate or multiwall carbon nanotubes, also induce flow limits in flame-retardant thermoplastics, so that the UL 94 classification is changed from dripping to non-dripping [19,20,21]. In contrast, melamine and its salts in polyamide promote dripping and are used to achieve dripping UL 94 classifications of V-2 and V-0, respectively [22,23]. Intumescent systems are believed to require an adjusted viscosity of the pyrolysing melt, which keeps the bubbles within the condensed phase, while enabling considerable deformation [24].

This work builds on previously-reported results [12,13] by relating the effects of different flame retardants in terms of the UL 94 test with the melt dripping behaviour of flame-retarded polymeric materials. Four polymers, poly(butylene terephthalate), (PBT HB (HB—horizontal burning test)), polypropylene (PP HB), heterophasic copolymer polypropylene modified with an ethylene-propylene rubber (PP-EP HB) and polyamide 6 (PA 6 HB), which showed an HB rating in the UL 94 test, as well as their flame retarded variations, for instance PBT V-2 (V—vertical burning test), PBT V-1, and PBT V-0 classified as V-2, V-1 or V-0, have been chosen. The selection of flame retarded polymers was also based on their distinctive flame-retardant mode of actions. Their melt dripping behaviours have been tested in a vertically-oriented furnace [12], and mass loss, drop size and drop temperatures were recorded. The viscosity, thermal transition, thermal and thermo-oxidative decomposition of the flame-retarded polymers and corresponding polymers have also been investigated. The results are analysed and compared to explain the different dripping behaviours and their relation to UL 94 classifications.

## 2. Results and Discussion

### 2.1. Thermal Transition, Thermal and Thermo-Oxidative Decomposition

Melt flow and dripping of flame-retarded polymers during exposure to an external heat source are a complex interaction of physical and chemical processes. The physical processes, such as glass transition and melting, as well as chemical, such as molecular weight reduction and charring, caused by thermal and thermo-oxidative decomposition play a major role with respect to melt flow and dripping. Thus, the effects of flame retardants on the thermal transition, thermal and thermo-oxidative decomposition of PBT HB, PBT V-2, PBT V-1, PBT V-0, PP HB, PP V-2, PP-EP HB, PP-EP V-2, PA 6 HB, PA 6 V-2 and PA 6 V-0 were investigated.

**Figure 1:**
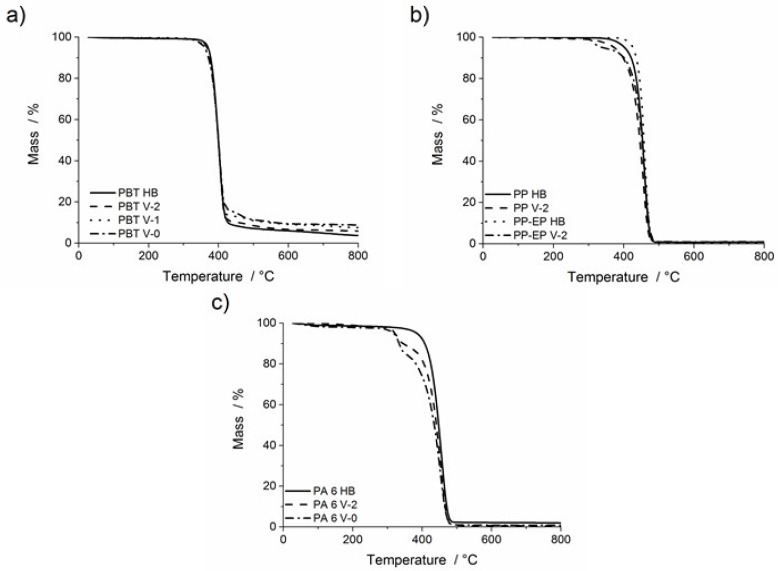
TGA under N_2_. Mass of: (**a**) poly(butylene terephthalate) (PBT) HB, PBT V-2, PBT V-1, PBT V-0; (**b**) polypropylene (PP) HB, PP V-2, PP-ethylene-propylene (EP) HB, PP-EP V-2; and (**c**) polyamide 6 (PA 6) HB, PA 6 V-2, PA 6 V-0. Heating rate: 10 °C·min^−1^.

The mass loss curves representing thermal decomposition (thermogravimetric analysis (TGA) under nitrogen atmosphere) of all investigated materials are given in Figure 1. The characteristic parameters of the thermal decomposition are presented in Table 1. PBT HB decomposed in one decomposition step, with a starting decomposition temperature (mass loss = 5 wt %) of *T*_5 wt %_ = 369 °C and a maximum mass loss rate (taken from the derivative thermogravimetric analysis (DTG)) at *T*_max_ = 398 °C. The mass loss of PBT HB at an endset temperature of *T*_end_ = 416 °C is 90.9 wt %. There is some steady subsequent decomposition, resulting in only 1.4 wt % remaining residue at 900 °C. The main decomposition pathway of PBT is due to a six-membered cyclic-activated complex with the decomposition products CO_2_, butadiene, tetrahydrofuran and terephthalic acid [25]. In contrast to PBT HB, the flame-retarded PBT V-2, PBT V-1 and PBT V-0 decomposed in two decomposition steps, starting from *T*_5 wt %_ = 368 °C for PBT V-2 and *T*_5 wt %_ = 360 °C for PBT V-1 and PBT V-0. The first main decomposition step of PBT V-2, PBT V-1 and PBT V-0 corresponds to the decomposition of the polymer with mass losses of 89, 86 and 82 wt %, respectively. A subsequent minor decomposition step started at 417 °C, which can be related mainly to the decomposition of the organic phosphinate in PBT [26], e.g., phosphinate moiety, ester carbonyl fragments and alkyl aromatic fragments [27,28]. The increase in residue above 400 °C is attributed mainly to aluminium phosphates increasing due to increasing concentrations of organic phosphinate (PBT V-2 < PBT V-1 < PBT V-0). The reduction in the first mass loss, the increase in the second mass loss and the increased residue at high temperatures correlated with the amount of flame retardant added.

**Table 1:** TGA: thermal decomposition.

Materials	*T*_5 wt %_	*T*_15 wt %_	1st Decomposition		2nd Decomposition		Residue at 900 °C
			Δ Mass Loss	*T*_max_	Δ Mass Loss	*T*_max_	
	/°C	/°C	/wt %	/°C	/wt %	/°C	/wt %
PBT HB	369	382	90.9	398	–	–	1.4
PBT V-2	368	381	89.0	405	4.6	–	3.1
PBT V-1	360	379	86.4	404	6.4	–	6.6
PBT V-0	360	377	82.6	404	7.8	447	8.3
PP HB	403	430	99.2	456	–	–	0.6
PP V-2	373	412	99.1	450	–	–	0.8
PP/EP HB	426	441	99.4	458	–	–	0.5
PP/EP V-2	339	417	99.1	455	–	–	0.9
			**Precedent Decomposition**		**Main Decomposition**		
PA 6 HB	383	415	–	–	97.3	458	1.3
PA 6 V-2	323	388	10.6	326	88.7	455	0.5
PA 6 V-0	323	353	14.0	336	85.6	453	0.4

Figure 1b shows the decomposition of PP HB, PP V-2, PP-EP HB and PP-EP V-2. PP HB shows a single decomposition step with *T*_max_ = 456 °C; *T*_5 wt %_ = 403 °C. PP-V-2 also decomposed in a single step, starting at a reduced *T*_5 wt %_ = 373 °C, showing a slightly reduced *T*_max_, and was completed at a similar *T*_end_ = 470 °C with a residue of 0.8 wt % remaining at the end of the experiment. The significant difference of 30 °C in the beginning of decomposition is proposed to be caused by a radical generator used as a flame retardant, enhancing the chain scission of PP. PP-EP HB undergoes single-step decomposition, whereas PP-EP V-2 decomposed in two steps (Figure 1b). The preceding minor decomposition is attributed to the decomposition of the brominated flame retardant tetrabromobisphenol A bis(2,3-dibromopropyl ether) (BDDP) with a decomposition temperature above 260 °C. Thus, *T*_5 wt %_ representing the decomposition of BDDP (Figure 1b) is lowered by 87 °C compared to PP-EP. The influence on the beginning of the main decomposition step of the PP matrix is apparent when *T*_15 wt %_ (=temperature at 15 wt % mass loss) is compared. The *T*_15 wt %_ = 417 °C of PP-EP V-2 occurred 24 °C earlier than the *T*_15 wt %_ = 441 °C for PP-EP HB, whereas *T*_max_ hardly changed. A similar earlier decomposition (∆*T* = 18 °C) is observed for *T*_15 wt %_ of PP V-2 compared to PP HB. Thus, both PP-EP V-2 and PP V-2 show particularly early decomposition. Taking into account that PP decomposes by random chain scission, it is assumed that early decomposition may also result in a crucial decreased molecular weight and, thus, may promote melt flow and dripping.

PA 6 HB decomposed in one step, as seen from Figure 1c. The decomposition started at *T*_5 wt %_ = 383 °C with *T*_max_ = 458 °C. The residue remaining after a maximum temperature of 900 °C was 1.3 wt %. In contrast, PA 6 V-2 and PA 6 V-0 decomposed in a two-step process, the first one being a minor decomposition step followed by the main decomposition of the polymer. Between *T*_5 wt %_ = 318 °C and 355 °C, a mass loss of 10.6 wt % for PA 6 V-2 and 14.0 wt % for PA 6 V-0, respectively, are attributed to the evaporation of melamine [22,29,30,31]. Above 320 °C, melamine cyanurate undergoes endothermic decomposition to melamine and cyanuric acid [22] and catalyses the dehydration of the primary amine chain ends. The difference in decomposition of PA 6 V-2 and PA 6 V-1 from PA 6 HB is exemplified by the change in *T*_15 wt %_. The *T*_15 wt %_ of PA 6 V-2 and PA 6 V-0 differed from PA 6 HB by 27 and 62 °C, respectively, and indicates an enhanced decomposition of the polymer chains by melamine cyanurate into chain fragments instead of the formation of caprolactam, resulting in a promotion of melt flow and dripping. The second main decomposition step of PA 6 V-2 and PA 6 V-0 with *T*_max_ = 453 °C and residues of 0.5 and 0.4 wt %, respectively, corresponds to the decomposition of the polymer.

**Figure 2:**
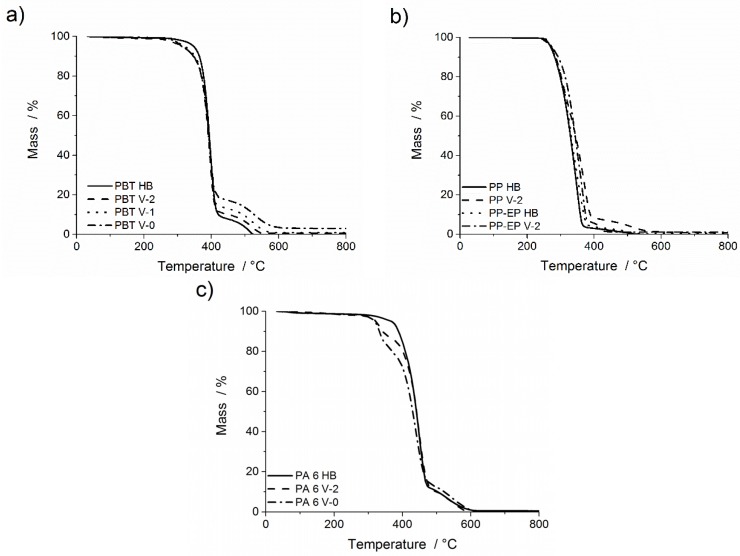
TGA under synthetic air. Mass of: (**a**) PBT HB, PBT V-2, PBT V-1, PBT V-0; (**b**) PP HB, PP V-2, PP-EP HB, PP-EP V-2; and (**c**) PA 6 HB, PA 6 V-2, PA 6 V-0. Heating rate: 10 °C min^−1^.

The TGA curves for thermo-oxidative decomposition of all materials under synthetic air are presented in Figure 2, and the analysed results are summarised in Table 2. PBT HB decomposed in a two-step decomposition process. The first main decomposition step between *T*_5 wt %_ = 347 and 411 °C with a mass loss of 90.5 wt % corresponds to the decomposition of PBT. The second subsequent minor decomposition step ending at *T*_end_ = 523 °C with a mass loss of 9.5 wt % is attributed to the oxidation of the char formed in the main decomposition step. No residue remained at *T* = 900 °C. PBT V-0 decomposed in two steps. The mass loss in the first main decomposition step was reduced (mass loss = 77.6 wt %) and started at lower temperatures (∆*T*_5 wt %_ = 35 °C), but showed a similar *T*_max_ = 396 °C. This corresponds to the decomposition of the polymer, part of which reacts with the flame retardant. The residue of the main step was increased and decomposed in a second subsequent decomposition and oxidation step (mass loss = 19.4 wt %). The temperature shift of this second step may be interpreted as increased stability due to a phosphorous cross-linked carbonaceous char structure. The small amount of residue at the end of the experiment may be attributed to aluminium phosphates [32,33]. The decomposition pattern of PBT V-2 and PBT V-1 lies in between those of PBT HB and PBT V-0, reflected by the increasing concentration of the aluminium diethyl phosphinate.

**Table 2:** TGA: thermo-oxidation.

Materials	T_5 wt %_	1st Decomposition		2nd Decomposition		3rd Decomposition		
		Δ Mass Loss	*T*_max_	Δ Mass Loss	*T*_max_	Δ Mass Loss	*T*_max_	Residue at 900 °C
	/°C	/wt %	/°C	/wt %	/°C	/wt %	/°C	/wt %
PBT HB	347	90.5	396	9.5	516	–	–	0.0
PBT V-2	325	88.7	394	11.3	511	–	–	0.7
PBT V-1	322	85.9	398	14.1	523	–	–	1.9
PBT V-0	312	77.6	396	19.4	529	–	–	3.1
PP HB	271	96.4	328	3.6	499	–	–	0.0
PP V-2	274	91.8	342	7.5	498	–	–	0.7
PP-EP HB	271	95.7	332	3.9	497	–	–	0.5
PP-EP V-2	272	93.3	344	5.7	495	–	–	1.2
PA 6 HB	368	–	–	88.1	449	11.5	529	0.4
PA 6 V-2	323	10.6	334	79.0	443	10.4	540	0.0
PA 6 V-0	318	14.0	332	73.2	441	12.8	553	0.0

Under thermo-oxidative conditions (Figure 2b), the major mass loss step for PP HB and PP V-2 started early, *T*_5 wt %_ = 270 °C, and, thus, differs clearly from the values obtained for thermal decomposition, by around ∆*T*_5 wt %_ = 100 °C for PP V-2 and 130 °C for PP HB. The start of thermo-oxidative decomposition of the polymer is no longer initiated by the radical generator. Above 200 °C, oxygen initiates a radical-chain decomposition of PP via hydroperoxidation of C–H bonds [34]. This thermo-oxidative decomposition also produces some carbonaceous residue for PP by oxidative dehydration. Starting at 361 and 382 °C, respectively, PP HB and PP V-2 exhibit a subsequent second decomposition step corresponding to the oxidation of the carbonaceous residue. PP V-2 shows an increase in the *T*_max_ of the main decomposition step and an increase in char value. Quite analogously, PP-EP HB and PP-EP V-2 decomposed in two decomposition steps, starting at *T*_5 wt %_ = 271 °C with a mass loss of 95.7 wt % and *T*_5 wt %_ = 272 °C with a mass loss of 93.3 wt %, respectively. The reduction of the decomposition temperature (*T*_5 wt %_) in synthetic air compared to nitrogen atmosphere is attributed to radical chain decomposition initiated by oxygen. After the subsequent minor decomposition step with *T*_max_ = 497 °C and a mass loss of 3.9 wt %, a residue of 0.5 wt % remained for PP-EP HB. The subsequent second decomposition step of PP-EP V-2 is characterised by *T*_max_ = 495 °C with a mass loss of 5.7 wt %.

Figure 2c shows the two-step decomposition of PA 6 HB, PA 6 V-2 and PA 6 V-0 in synthetic air. The *T*_5 wt %_ is similar to the value under nitrogen atmosphere, and the effect of the flame retardant is still noticed. The main decomposition step of PA 6 HB, starting at *T*_5 wt %_ = 368 °C, is due to the decomposition of the polymer chains with a mass loss of 88.1 wt %. The subsequent second step with a mass loss of 11.5 wt % is attributed to the oxidation of char residue. PA 6 V-2 and PA 6 V-0 decomposed in a three-step process. PA 6 V-2 and PA 6 V-0 exhibited a preceding first decomposition step due to decomposition of the melamine cyanurate. This step started at *T*_5 wt %_ = 323 °C for PA 6 V-2 and *T*_5 wt %_ = 318 °C for PA 6 V-0, respectively, with a mass loss representing the flame retardant content in the polymer. The mass loss of >70 wt % in the second (main) step was due to the decomposition of the polymer. The third subsequent decomposition step of PA 6 V-2 and PA 6 V-0 equals the second step of PA 6 HB and corresponds to the oxidation of the char residue of PA 6. The maximum temperature of the third decomposition is noted as *T*_max_ = 553 °C and *T*_max_ = 540 °C for PA 6 V-2 and PA 6 V-0, respectively. At a temperature of 900 °C, no residue remained.

The glass transition and melting temperatures were measured using differential scanning calorimetry (DSC) (Figure S1, Table 3). No noticeable influence of the flame retardants on the glass transition and melting temperature of the respective polymers was observed. PBT HB, PBT V-2, PBT V-1 and PBT V-0 exhibited similar glass transition temperatures and melting temperatures at around 53 and 225 °C, respectively. The melt enthalpies of PBT V-2, PBT V-1 and PBT V-0 compared to PBT HB decreased, but not as much as expected due to the flame retardant content. Most probably an increase in PBT crystallinity occurred due to the flame retardant particles working as a nucleating agent for crystallization. The characteristic temperatures of PP HB, PP V-2, PP-EP HB and PP-EP V-2 were glass transition at about −10 °C and −7 °C, respectively, and melting at 157 °C. The melt enthalpy for PP-EP was lower than for PP according to the EP content. The melt enthalpy values of PP V-2 and PP-EP V-2 were not changed compared to PP HB and PP-EP HB. PA 6 HB, PA 6 V-2 and PA 6 V-0 displayed the same glass transition temperature at 56 °C and a melting temperature at 221 °C. PA 6 V-2 and PA 6 V-0 showed a decrease in melt enthalpy, most probably also less than expected from the flame retardant content.

**Table 3:** Thermal transition (DSC).

Materials	Glass Transition Temperature	Melting Temperature	Enthalpy
	/°C	/°C	/J·g^−1^
PBT HB	55	225	50
PBT V-2	51	225	48
PBT V-1	51	224	48
PBT V-0	53	225	47
PP HB	−7	157	84
PP V-2	−7	157	85
PP-EP HB	−10	156	71
PP-EP V-2	−9	157	70
PA 6 HB	54	221	63
PA 6 V-2	56	220	61
PA 6 V-0	56	221	59

In the investigated systems, there is a limited overall influence of the flame retardants used on the thermal transition (glass transition and melting), thermal decomposition (thermal analysis under nitrogen) and thermo-oxidative decomposition (thermal analysis under air). The only significant change is at the beginning of decomposition, indicating an enhancement of decomposition caused by the flame retardants. Particularly, the thermal decomposition of PP, PP-EP and PA 6 is enhanced at the beginning of decomposition, as is the thermo-oxidative decomposition of PBT and PA 6. Even though the change in mass release is limited to the beginning of the decomposition, one can expect an impact on the viscosity of the pyrolysing melt of PP and PA 6 responsible for enhancing the melt flow and dripping behaviour. In PBT samples, additional residue and char formation occurred, which may also influence the viscosity.

### 2.2. Melt Dripping

**Figure 3:**
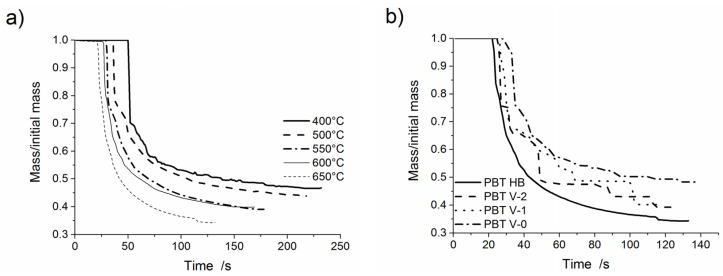
Mass loss as a function of time for: (**a**) PBT HB at various furnace temperatures; and (**b**) PBT HB, PBT V-2, PBT V-1 and PBT V-0 at 650 °C.

**Figure 4:**
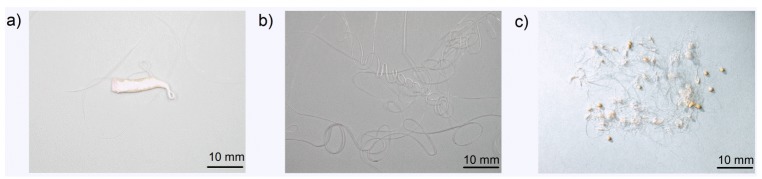
(**a**) Partly molten piece falling down of PBT V-2 at a furnace temperature of 500 °C; (**b**) melt flow material of PBT HB at 400 °C; (**c**) melt flow and dripping of PBT HB at 650 °C.

The melt dripping behaviour of all polymers was investigated by exposing polymer bars to convective heat in a purpose-built tube furnace, preheated to a set temperature. Each polymer and flame-retarded polymer was placed in the furnace at four/five different temperatures, which were selected in the temperature range between the temperature at which melt dripping starts and the temperature at which the sample ignites and starts burning. The mass loss due to volatilization and melt dripping as a function of time was recorded for each sample. The starting time of mass loss declines with increasing furnace temperature due to the faster melt dripping and volatilisation. The mass difference in residue is attributed to the remainder of polymer bar on the hook above the furnace. Since half of the sample was in the heated centre of the furnace, the residues are between 35% and 60%. Due to an increased furnace temperature, more of the polymer bar above the furnace was melted and contributed to dripping. Figure 3a shows the mass loss curves of PBT HB at various furnace temperatures. After an abrupt mass loss due to the first only partly molten piece falling down (Figure 4a), all materials showed a steady mass loss due to melt flow and dripping. The remaining material decreases with increasing furnace temperature. Mass loss curves for PBT HB, PBT V-2, PBT V-1 and PBT V-0 at 650 °C are shown in Figure 3b and summarised in Table 4. The mass loss due to dripping decreased from 60.2% ± 0.2% for PBT HB down to 39.7% ± 0.6% for PBT V-0. The mass loss caused by volatilisation increased by 8.5%–13.1% ± 0.8% for PBT V-0. The residue over the furnace increased in the order PBT HB < PBT V-2 ≤ PBT V-1 < PBT V-0. The starting time for mass loss was delayed, so melt dripping and, hence, the mass loss rate declined with increasing UL 94 classification. At 400 °C, the time until the first drop fell was 50 s for PBT HB, 66 s for PBT V-2, 70 s for PBT V-1 and 88 s for PBT V-0. The delay in dripping was attributed to the PTFE adjuvant working as an anti-dripping agent. The first drop is rather a break-up of the bottom part of the specimen and consisted of molten and non-molten material (Figure 4a). After this first break-up, PBT HB showed a constant melt flow (no drops; Figure 4b) for furnace temperatures <650 °C and tiny drops (average diameter 0.9 mm; Figure 4c) for ≥650 °C. The average drop diameter of PBT V-2, PBT V-1 and PBT V-0 was 11 mm.

**Table 4:** Summary of melt dripping results for PBT HB, PBT V-2, PBT V-1 and PBT V-0 at 650 °C.

Sample	No. of Drops	Total Mass Loss Due to Dripping	Total Mass Loss Due to Volatilization	Residual Mass
		/%	/%	/%
PBT HB	63 ± 3	60.2 ± 0.2	4.6 ± 0.7	35.2 ± 1.5
PBT V-2	25 ± 3	50.9 ± 2.8	7.6 ± 3.2	39.5 ± 0.4
PBT V-1	26 ± 2	50.1 ± 0.8	8.3 ± 2.4	41.6 ± 1.6
PBT V-0	25 ± 1	39.7 ± 0.6	13.1 ± 0.8	47.2 ± 0.2

**Figure 5:**
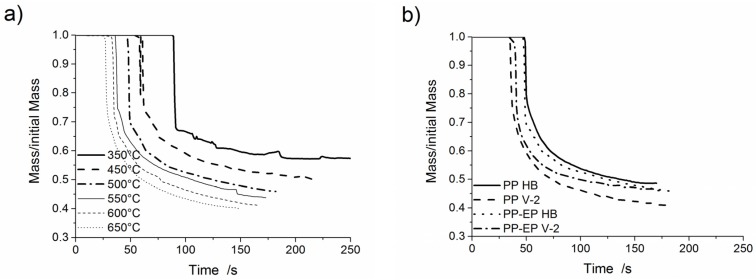
Mass loss as a function of time of: (**a**) PP-EP HB at various furnace temperatures; and (**b**) PP HB, PP V-2, PP-EP HB and PP-EP V-2 at 500 °C.

**Figure 6:**
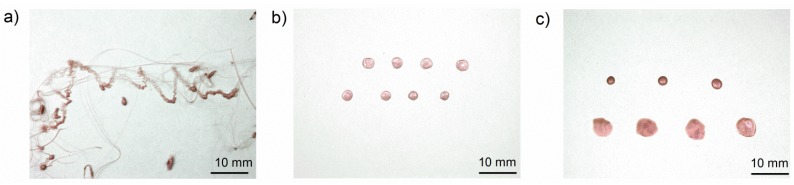
(**a**) Melt flow and dripping of PP-EP HB at a furnace temperature of 450 °C; (**b**) drops of PP-EP V-2 at 500 C; and (**c**) drops of PP-EP V-2 at 600 °C.

Figure 5a presents mass loss curves of PP-EP HB at various furnace temperatures. Figure 5b shows the mass loss curves of PP-EP HB, PP-EP V-2, PP HB and PP V-2 at a 500 °C furnace temperature. The dripping characteristics, e.g., number of drops, total mass loss due to dripping, total mass loss due to volatilization and residual mass, are summarised in Table 5. PP HB, PP V-2, PP-EP HB and PP-EP V-2 showed an abrupt mass loss due to the first partly molten piece falling down. The subsequent melt dripping behaviours in different samples differ depending on the flame retardant type and concentration. An earlier mass loss and higher total mass loss in PP V-2 compared to PP HB and in PP-EP V-2 compared to PP-EP HB, respectively, go well together with the enhanced decomposition (reduction of *T*_5 wt %_) in TGA measurements. Both flame-retarded materials, PP V-2 and PP-EP V-2, showed an increase in the mass loss due to dripping. The mass loss caused by volatilisation decreased from 7.4% ± 1.3% and 7.5% ± 1.6% for PP HB and PP-EP HB, respectively, down to 4.5% ± 1.5% for PP V-2 and 4.2% ± 0.7% for PP-EP V-2. PP-EP HB and PP-EP V-2 began dripping after 51 and 40 s, respectively, at a 500 °C furnace temperature; the total mass loss of PP-EP V-2 was reduced. PP V-2 exhibited a similar decrease in the starting time of mass loss and an increase in total mass loss of 10 wt %. PP-EP HB showed a constant melt flow with no drops. In contrast, PP-EP V-2 exhibited tiny drops (average drop diameter: 1.2 mm; PP-EPV-2 drops shown in Figure 6a) in quick succession at lower temperatures. At 500 °C, the size and frequency of PP-EP V-2 drops changed to medium-sized (5 mm, presented in Figure 6b), occasional drops. The PP-EP V-2 drop succession (Figure 6c) on the aluminium foil at 600 °C showed a size and shape variation from an initial 10-mm diameter with an irregular shape to circular drops 4 mm in diameter.

**Table 5:** Summary of melt dripping results for PP HB, PP V-2, PP-EP HB and PP-EP V-2 at 500 °C.

Sample	No. of Drops	Total Mass Loss Dripping	Total Mass Loss Volatilization	Reside Mass
		/%	/%	/%
PP HB	44 ± 3	41.3 ± 0.6	7.4 ± 1.3	51.3 ± 0.7
PP V-2	77 ± 8	53.9 ± 0.3	4.5 ± 1.5	41.6 ± 1.2
PP-EP HB	43 ± 2	41.6 ± 0.8	7.5 ± 1.6	50.9 ± 0.8
PP-EP V-2	75 ± 5	50.7 ± 0.2	4.2 ± 0.7	45.1 ± 0.5

**Figure 7:**
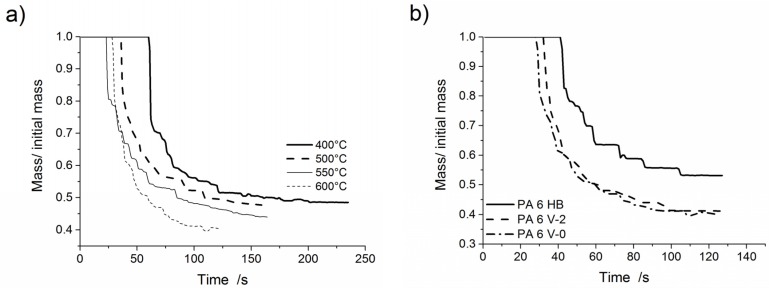
Mass loss as a function of time for: (**a**) PA 6 V-2 at various furnace temperatures; and (**b**) PA 6 HB, PA 6 V-2 and PA 6 V-0 at 600 °C.

**Figure 8:**
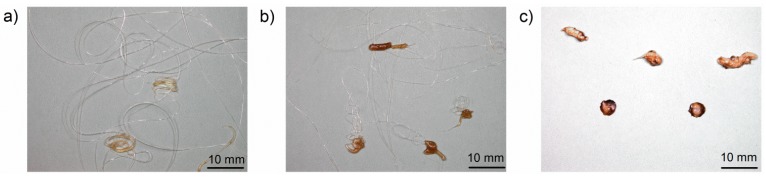
(**a**) Melt flow of PA 6 HB at a furnace temperature of 400 °C; (**b**) melt flow and drops of PA 6 HB at 550 °C; and (**c**) drop pathway of PA 6 V-2 at 550 °C.

Mass loss curves of PA 6 V-2 at various furnace temperatures are shown in Figure 7a. Figure 7b presents mass loss curves for PA 6 HB, PA 6 V-2 and PA 6 V-0 at a furnace temperature of 600 °C. The main results are summarised in Table 6. PA 6 HB, PA 6 V-2 and PA 6 V-0 exhibited an abrupt mass loss due to the first partly molten piece falling down followed by melt dripping. PA 6 V-2 and PA 6 V-0 showed a very similar mass loss. The total mass loss and starting time of the mass loss were affected by the flame retardant. The total mass of PA 6 V-0 to PA 6 HB varied by 15 wt %. Mass loss due to dripping was nearly doubled to 45.3% ± 1.3% for PA 6 V-2 and 47.4% ± 1.6% for PA 6 V-0. The mass loss caused by volatilisation halved from 22.1% ± 2.5% for PA 6 HB to a value of 12.1% ± 1.8% and 11.9% ± 2.4% for PA 6 V-2 and PA 6 V-0, respectively. At a 600 °C furnace temperature, PA 6 HB and PA 6 V-0 began dripping after 41 and 28 s of exposure, respectively. The reduced starting time, in combination with higher total mass loss, is attributed to a decrease in the viscosity of PA 6 due to melamine cyanurate enhancing the decomposition, as was observed in the TGA experiments. PA 6 HB exhibited a constant, but low melt flow at low temperatures (Figure 8a). At 550 °C, the material developed medium-sized drops (average diameter 6 mm; Figure 8b) concurrent with a low melt flow. The shapes of the drops of PA 6 V-2 and PA 6 V-0, shown in Figure 8c, varied from irregular to circular at 550 °C. The colours of the drops varied from white to light to dark brown. This suggests partial decomposition of the molten drop residues.

**Table 6:** Summary of melt dripping results for PA 6 HB, PA 6 V-2 and PA 6 V-0 at 600 °C.

Sample	No. of Drops	Total Mass Loss Dripping	Total Mass Loss Volatilization	Reside Mass
		/%	/%	/%
PA 6 HB	32 ± 4	23.9 ± 1.3	22.1 ± 2.5	54.0 ± 1.2
PA 6 V-2	40 ± 2	45.3 ± 0.5	12.1 ± 1.8	42.6 ± 1.3
PA 6 V-0	47 ± 1	47.5 ± 1.6	11.9 ± 2.4	40.7 ± 0.8

### 2.3. Temperatures of Melting Drops

**Figure 9:**
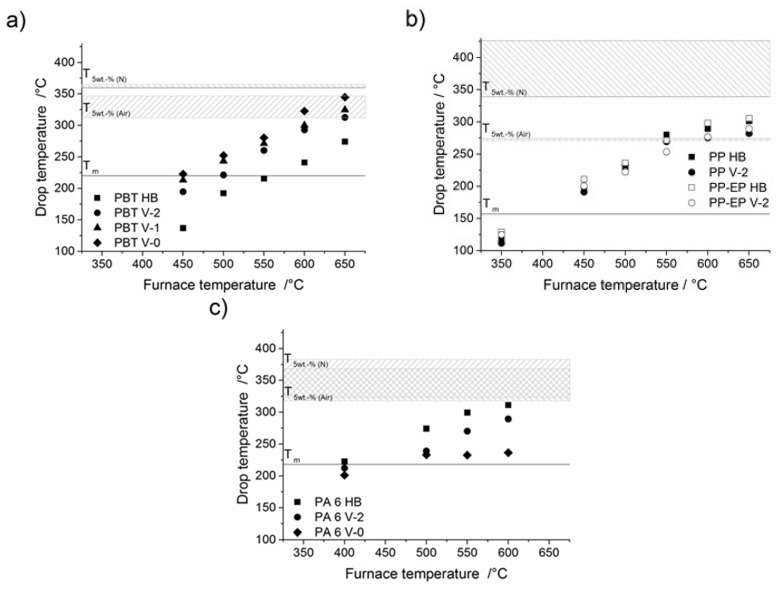
Temperature of drops as a function of furnace temperature with various UL 94 classifications for: (**a**) PBT HB, PBT V-2, PBT V-1, PBT V-0; (**b**) PP HB, PP V-2, PP-EP HB, PP-EP V-2; and (**c**) PA 6 HB, PA 6 V-2, PA 6 V-0.

To measure the temperatures of the drops, the experimental setup is similar to that used for melt dripping, *i.e.*, the test bars are fixed, and the pre-heated furnace is raised in position. Similar to the phenomenon in fire tests, such as vertical UL 94, the material escapes from further heating in the furnace by dripping. This study was undertaken to relate the temperatures of the molten drops to the decomposition temperatures (*T*_5 wt %_ from TGA) of the respective polymers in order to get an indication of the degree of decomposition. The drop temperatures are average temperatures recorded by drops falling directly on and through circularly-arranged thermocouples immediately beneath the furnace. The setup delivered quite accurate results with an uncertainty of only ±2 °C. The drop temperatures of PBT, PP, PP-EP and PA 6 with various UL 94 classifications as a result of different furnace temperatures are plotted in Figure 9. The drop temperatures were much lower than the set furnace temperatures. Thus, the main response of the test bar to an external heating source was melting and dripping; the thermal decomposition of the material is a minor effect that became more important at higher temperatures. The temperature of the first only partly molten piece falling down was about 50 °C for PP, PP-EP and PBT and 100 °C for PA 6 at all furnace temperature settings, which is much lower than the temperatures recorded for the subsequent drops given in Figure 9, and this was disregarded for the calculation of the average drop temperature at a particular furnace setting. The melting temperatures, as well as the beginning of decomposition measured by DSC and TGA (Table 3) are also presented as horizontal lines and bars, respectively, in Figure 9.

Dripping below the melt temperature is mainly a result of local softening of the specimen. All polymers independent of the exposed temperature showed this behaviour at the first partly molten piece falling down. Above the melting temperature, ordered crystalline lattice domains melt into an unordered liquid. This transition from a thermoelastic to a thermoplastic state is associated with a loss in mechanical properties. Between melting temperature and decomposition temperature, the viscosity of the material decreases, and the polymer starts melt flowing and dripping. Beyond the decomposition temperature (*T*_5 wt %_), polymers start to decompose. At this stage, the drops consisted of both molten and decomposed polymer.

Figure 9 presents the drop temperatures of PBT HB, PBT V-2, PBT V-1 and PBT V-0 as a function of the furnace temperature. The average drop temperature of all PBT compounds increased linearly between 200 °C and the starting temperature of decomposition. The drop temperature of PBT HB at the lowest furnace temperature of 450 °C was very low and far below the measured melting temperature in the DSC. The break-up of only partly molten materials was concluded to be similar to that for the first drops. PBT V-2, PBT V-1 and PBT V-0 exhibited similar drop temperatures much closer to the expected melting temperature at a furnace setting of 450 °C. For all of the investigated furnace temperatures, the drop temperatures were observed in the order PBT HB << PBT V-2 < PBT V-1 < PBT V-0. At a furnace temperature of 650 °C, the observed drop temperatures of PBT HB were about 274 and 312 °C for PBT V-2, 324 °C for PBT V-1 and 344 °C for PBT V-0, respectively. The drop temperatures of PBT V-2 are always 30–50 °C higher than the drop temperatures of PBT HB. The drop temperatures of PBT V-2 < PBT V-1 < PBT V-0 were observed in margins of 20–30 °C. The drop temperatures agree very well with the mass loss results (Figure 3b). With higher UL 94 classification, a delay in mass loss occurred, associated with higher drop temperatures.

PP HB, PP V-2, PP-EP HB and PP-EP V-2 exhibited drop temperatures below the melting temperature at the lowest furnace setting at 350 °C. Both an insufficient material quantity due to a low melt flow and the distance between furnace and thermocouples might be the cause of these drop temperatures. The average drop temperature of PP HB, PP V-2, PP-EP HB and PP-EP V-2 increased linearly with increasing furnace temperature up to the temperature at the beginning of decomposition (*T*_5 wt %_). Above this temperature, the drop temperatures showed a clear kink and approximated a plateau at about 310 °C, shown in Figure 9b. The kink in the dependence of drop temperature on furnace temperature indicates decomposition of the polymer. The flame retardant in PP V-2 and PP-EP V-2 caused a reproducible reduction of the drop temperature in comparison to the drop temperature of PP HB and PP-EP HB, respectively. The drop temperatures of PP V-2 and PP-EP V-2 to PP HB and PP-EP V-2 differed by 17 ± 3 °C at higher furnace temperatures. The drop temperatures correspond very well to the starting time of mass loss in the dripping experiment (Figure 5b), as well as to decomposition temperature (*T*_5 wt %_). The earlier mass loss in the dripping experiment goes along with the reduction of the drop temperature. The decomposition temperature and the associated shift of the decomposition product balance towards low molecular chain fragments limit the maximum drop temperature. The dramatic reduction of the complex viscosity supports the drop temperature results.

Figure 9c shows the drop temperature of PA 6 HB, PA 6 V-2 and PA 6 V-0. The drop temperatures of PA 6 HB increase rather linearly with increasing furnace temperatures until the decomposition temperatures (*T*_5 wt %_) are reached. The drop temperature levelled off at the decomposition temperature at a furnace temperature of 600 °C. The drop temperatures were observed in the order PA 6 HB > PA 6 V-2 > PA 6 V-0. At furnace temperatures of 500 and 550 °C, the drop temperature of PA 6 V-2 is around 30 °C lower than the drop temperature of PA 6 HB. The drop temperatures of PA 6 V-0 levelled off at around 233 °C, a temperature far below the onset of the decomposition temperature. Thus, with increasing furnace temperature, the drop temperature of PA 6 V-0 exhibited drop temperatures far below PA 6 HB and PA 6 V-2. The decrease in drop temperature for PA 6 V-2 and PA 6 V-0 is proposed to be directly linked to the earlier dripping and to be caused by the enhanced decomposition compared to PA 6 HB.

### 2.4. Rheological Measurement

Of course, the dripping experiments indicated that the viscosity, of the melt and the pyrolysing melt, is one of the most important factors controlling the dripping behaviour. Thus, rheological experiments on the polymers, as well as the collected drops were performed under well-defined conditions.

**Figure 10:**
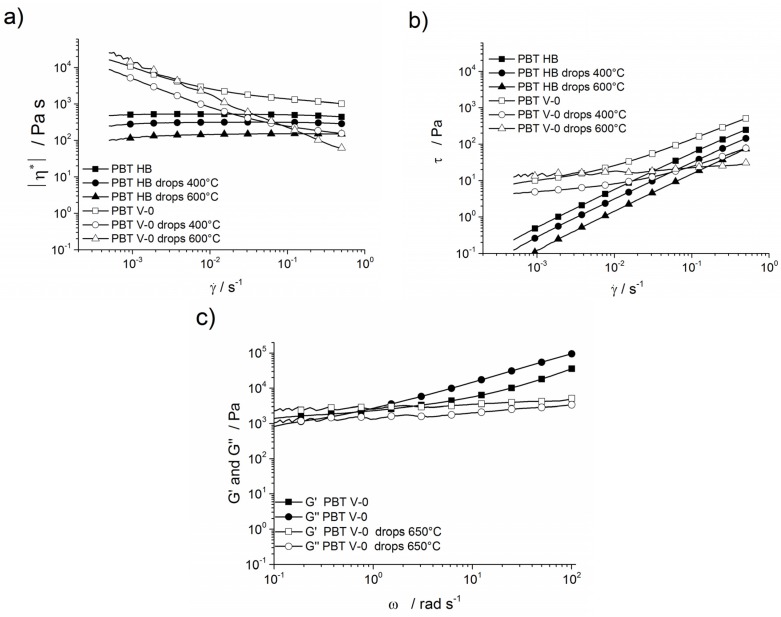
(**a**) Complex viscosity of PBT HB, PBT V-0 and their drops as a function of shear rate; (**b**) shear stress *versus* shear rate for PBT HB, PBT V-0 and its drops; and (**c**) storage modulus and loss modulus of PBT V-0 and its molten drops at 650 °C.

Figure 10a shows the complex viscosity of PBT HB, PBT V-0 and of the corresponding drops collected in the dripping experiment. The complex viscosity (│η*│) curve of PBT HB is rather horizontal, increasing only slightly at higher shear rates ($\dot{\text{γ}}$). The complex viscosity of PBT HB drops collected in the experiments at furnace temperatures of 400 and 600 °C is reduced compared to that of the PBT HB. The reduction is more pronounced with increasing furnace temperature and can be attributed to partial decomposition of the polymer, where shorter chains resulted in lower viscosities. In contrast, the complex viscosity of PBT V-0 showed a plateau only for higher shear rates and a clear increase with decreasing shear rate. The increase was linear in the log-log plot. Thus, PBT V-0 showed a solid-like behaviour at low shear rates. The investigated drops showed a similar behaviour at low shear rates and a decrease in the plateau viscosity even more pronounced than that observed for PBT HB. The greater reduction in the plateau for the viscosity corresponds to the increased drop temperatures for PBT V-0 (Figure 9a). Thus, the change for PBT V-0 between viscosity increases with decreasing shear rates and plateau behaviour for higher shear rates shifted to higher shear rates; for the drop collected at the 600 °C furnace temperature, only a linear decrease was observed. For unlinked polymers, the pertinent viscosity for dripping is consistent with the ordinary steady-flow viscosity [35]. Thus, in vertical UL 94 conditions, melt flow and dripping occur at low shear rates. In Figure 10b, the shear rates are shown as a function of shear stress. Without flame retardant, the shear stress of PBT HB and its drops decreases linearly with decreasing shear rates. The lines are parallel to each other; the shear stress is reduced for the drops. At higher furnace temperatures, the shear stress of the drops of PBT V-0 decreases due to partial decomposition of the polymer chains. The PBT V-0 curve exhibits a limited decrease in shear stress at low shear rates. The change in the behaviour between higher and lower shear rates is marked by a yield point. The certain resistance to stresses at low shear rates belongs to a solid-like behaviour and indicates a flow limit; *i.e.*, melt flow and dripping are hindered. This effect due to the addition of PTFE as an anti-dripping agent [10] is perceptible in PBT V-2, PBT V-1, PBT V-0 and its drops. With increasing furnace temperatures and, thus, increasing decomposition, the yield point shifts to higher shear rates. The reduced melt flow resulted in the formation of larger drops with higher drop temperatures. Figure 10c shows the storage modulus *G*′ and the loss modulus *G*″ as functions of the angular frequency of PBT V-0 and its drops at a 650 °C furnace temperature. For the frequency-dependent values storage (*G*′) and loss (*G*″) moduli, the melt characteristics, e.g., frictional forces and elasticity, are important. The storage and loss modulus curves of PBT V-0 display a viscoelastic behaviour dominated by viscous liquid behaviour (*G*′ < *G*″ cross-over frequency: 0.4 rad·s^−1^). A greater storage modulus *G*′ than loss modulus *G*″ (*G*′ > *G*″) indicates a solid-like state [36]. Only the solid-like behaviour of the PBT V-0 drops above the decomposition temperature (*T*_5 wt %_) with no cross-over frequency being observed, and this can be attributed to the reduced matrix impact due to chain decomposition, as well as the enhanced evolution of a polyaromatic char structure and terephthalic acid in the condensed phase by organic phosphinate [37].

**Figure 11:**
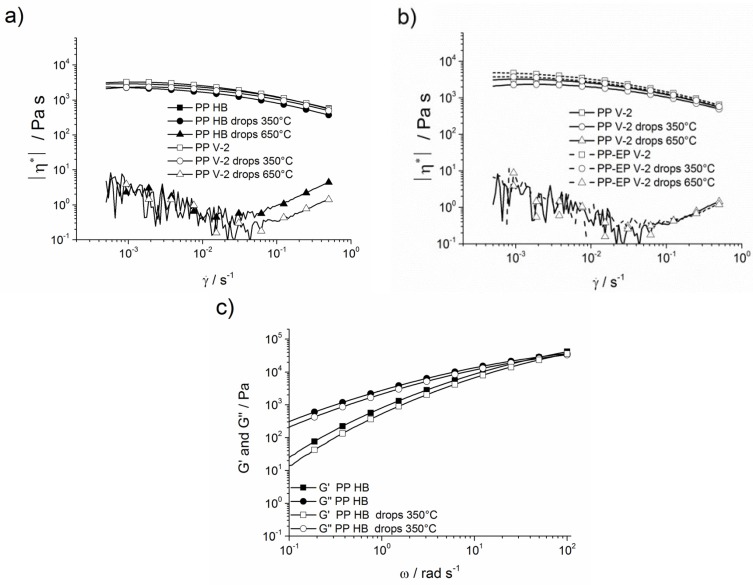
(**a**) Complex viscosity of PP HB, PP V-2 and their molten drops as a function of shear rate; (**b**) complex viscosity of PP V-2, PP-EP V-2 and their molten drops as a function of shear rate; and (**c**) storage modulus and loss modulus of PP HB and its molten drops at 350 °C.

The rheological behaviour of PP HB and PP V-2 and its drops is presented in Figure 11a. The complex viscosity showed a plateau value at low shear rates and is consistent with the zero-shear viscosity. With increasing shear rates, all materials, except drops, exposed to a 650 °C furnace temperature showed a decrease in complex viscosity due to a reduction of lost deformation energy. No significant variation in the curve shape between PP V-2, PP HB and their respective drops exposed to a 350 °C furnace temperature was noted. PP V-2 and its drops at 350 °C had slightly higher viscosity than PP HB. For both PP HB and PP V-2, a tiny decrease in complex viscosity was ascertained for the drops at just a 350 °C furnace temperature, caused by partial decomposition of the polymer chains. The complex viscosity decreased dramatically and actually could no longer be properly determined (the data appear to be noisy, indicating the loss of reliability) for PP HB and PP V-2 drops at a furnace temperature of 650 °C. The viscosity in combination with drop temperatures above the decomposition temperature (*T*_5 wt %_) indicated fairly complete damage of the polymer chain structure due to random chain scissions [38]. PP-EP displayed a behaviour similar to PP, shown in Figure 11b. The decreased complex viscosity of PP-EP V-2 drops exposed to a 350 °C furnace temperature indicates the initial decomposition. The PP-EP V-2 drops at a 650 °C furnace temperature showed a very similar viscosity behaviour, indicating intensive decomposition as PP V-2 drops exposed to a 650 °C furnace temperature. Figure 11c shows the storage and loss moduli of PP HB and its drops exposed to 350 °C. PP HB and its drops displayed a *G*′ < *G*″ behaviour. The cross-over frequency of PP HB is noted at 53.4 rad·s^−1^, with a value of 61.4 rad·s^−1^ for its drops.

**Figure 12:**
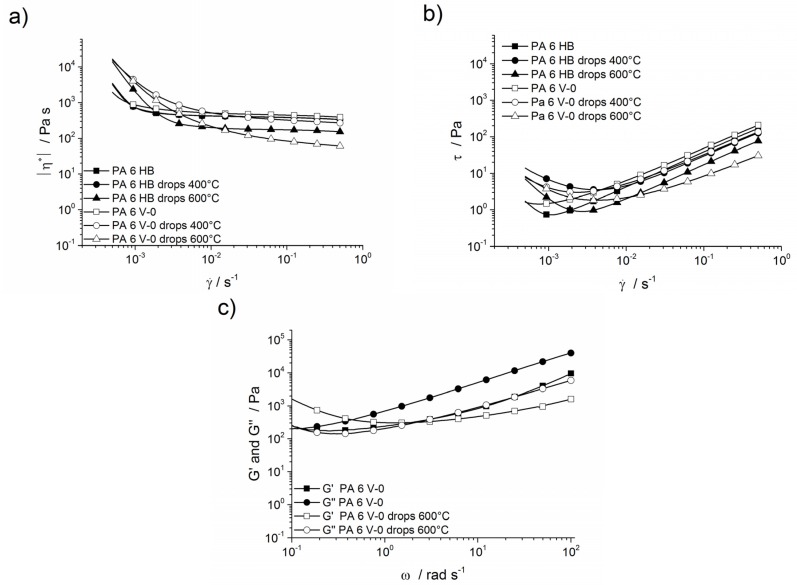
(**a**) Complex viscosity of PA 6 HB, PA 6 V-0 and their drops as a function of shear rate; (**b**) shear stress *versus* shear rate for PA 6 HB, PA 6 V-0 and its drops at a 600 °C furnace temperature; and (**c**) storage modulus and loss modulus of PA 6 V-0 and its drops at a 600 °C furnace temperature.

The complex viscosities for PA 6 HB, PA 6 V-2, PA 6 V-0 and their drops at 400 and 600 °C are shown in Figure 12a. The rheology was measured on samples stored at 23 °C and 50% humidity, so that all samples show an increase in viscosity and resistance towards flow (Figure 12b) at low shear rates due to their water content and corresponding H-bonding. The increased complex viscosity at low shear rates is caused by an increase in the storage modulus, shown in Figure 12c. The PA 6 HB, PA 6 V-0 and its drops, except for PA 6 V-0, exposed to a 600 °C furnace temperature, exhibited a constant complex viscosity at higher shear rates. The cross-over frequency of PA 6 V-0 is noted at 0.1 rad·s^−1^. The drop material of PA 6 V-0 exposed to 400 and 600 °C had a cross-over frequency at 0.7 and 2.3 rad·s^−1^, respectively. The material therefore has a dominant elastic behaviour at low shear rates. The shear stress decreased with increasing shear rate, approximated a limit and increased at higher shear rates. This phenomenon is dependent on the shear rate and the time, *i.e.*, the drying effect [39] of the sample during the measurement. The viscosity values at low shear rates may not reflect the actual dripping behaviour of the materials. The complex plateau viscosity is defined as Newtonian viscosity and is discussed to compare the viscosity during melt flow and dripping. PA 6 V-0 had a slightly higher viscosity than PA 6 HB due to melamine cyanurate particles. The plateau viscosity value of PA 6 V-0 was about 500 Pa·s, with 400 Pa·s for PA 6 HB and 300 and 300 Pa·s for their drops obtained on exposure of the polymers to a 400 °C furnace temperature. This reduction in complex viscosity is attributed to the initiated decomposition of the polymer chains. The complex viscosity of PA 6 V-0 drops exposed to a furnace temperature of 400 °C exhibited a higher complex viscosity than did PA 6 HB drops. The products of the self-condensation of melamine help the polymer to remain in the condensed phase, to undergo charring [22] and to build up a solid phase. Increasing char residue formation is also noted in the TGA. Under synthetic air, the mass loss of the last decomposition step increased along with flame-retarding content (PA 6 HB 11.5 wt %, PA 6 V-0 14.1 wt %). The drops of PA 6 HB and PA 6 V-0 exposed to 600 °C showed a viscosity reduction at higher shear rates due to advanced polymer decomposition. In comparison to the viscosity of PA 6 HB drops at 600 °C, the decreased and more sloped complex viscosity of PA 6 V-0 drops at a 600 °C furnace temperature indicates an increased decomposition towards smaller chain fragments due to melamine cyanurate.

### 2.5. Mode of Actions of the Flame Retardant in the UL 94 Test

The halogen-free flame retardant in combination with PTFE in PBT increased the viscosity at low shear rates by an induced flow limit for shear rates below a yield point. The certain resistance to stresses at low shear rates hinders melt flow and dripping. The drop temperature, drop size and starting time of mass loss increased, whereas the total mass loss decreased. The V-0 classified material PBT V-0 was non-dripping in the UL 94 test.

Tetrabromobisphenol A bis(2,3-dibromopropyl ether) in PP-EP V-2 decreased the *T*_5 wt %_ decomposition temperature, the starting time of mass loss in the dripping experiment, the drop temperature, the drop size and the viscosity of the PP-EP V-2 drops. The total mass loss in the dripping experiment increased. All of these factors correspond to each other consistently and indicate an enhanced decomposition of the polymer chains by the halogenated flame retardant and, thus, promoted dripping. PP-EP V-2 achieved a V-2 classification in the UL 94 test due to enhanced dripping of the polymer and removal of fuel and heat from the flame zone. The flame retardant in PP V-2 functioned similarly and enhanced the dripping of the polymer by shifting the balance of the decomposition product to a lower molecular mass.

In the dripping experiment, melamine cyanurate decreased the starting time of mass loss, the *T*_5 wt %_ decomposition temperature and the drop temperature in PA 6 V-2 and PA 6 V-0. These reductions suggest an enhanced decomposition to low molecular decomposition chain fragments associated with the promotion of dripping. The complex viscosity of drops of PA 6 V-0 at a 600 °C furnace temperature decreased. This relationship confirms the interference of melamine cyanurate in the decomposition of PA 6. PA 6 V-2 and PA 6 V-0 achieved a V-2 and V-0 classification in the UL 94 test due to enhanced dripping of the polymer and removal of fuel and heat from the flame zone, respectively.

## 3. Experimental Section

### 3.1. Materials

The commercial flame retardant PBT V-0 (Celanex XFR 4840, Celanese, Frankfurt, Germany) contains organic phosphinate and had a UL 94 classification of V-0. PBT with UL 94 Classifications V-2 (PBT V-0) and V-1 (PBT V-1) was compounded from HB-classified PBT (PBT HB, Celanex 2002-2, Celanese, Frankfurt, Germany) with 5% and 10% aluminium diethyl phosphinate (Exolit OP1240, Clariant, Sulzbach, Germany), respectively. PBT V-2, PBT V-1 and PBT V-0 contained a small amount of PTFE.

The flame retardant PP V-2 (Polyflam RPP 2000, A. Schulmann, Kerpen, Germany) contains a halogen-free, radical-generating flame retardant and has a UL 94 classification of V-2. Non-flame-retarded PP with UL 94 classification of HB was provided by LyondellBasell (PP HB, Moplen HP501H, A. LyondellBasell, Frankfurt, Germany). PP-EP V-2 (Hostacom PPR 7342 FL, LyondellBasell, Frankfurt, Germany) contained a halogen-containing flame retardant, BDDP and Sb_2_O_3_. The UL 94 classification was V-2. PP-EP with the same impact modification as PP-EP V-2 (PP-EP HB, Moplen EP 300K, LyondellBasell, Frankfurt, Germany) was used non-flame-retarded with UL 94 Classification HB.

PA 6 was used in three UL 94 classifications: HB (PA 6 HB, Technyl C216H2 Natural, Rhodia, Freiburg, Germany), V-2 (PA 6 V-2, Technyl C206F Natural, Rhodia, Freiburg, Germany) and V-0 (PA 6 V-0, Technyl C50H2 Natural, Rhodia, Freiburg, Germany), respectively. PA 6 V-0 and PA 6 V-2 contain melamine cyanurate. All materials, displayed in Table 7, were obtained in the form of polymer pellets.

**Table 7:** Material.

Materials	UL 94 Classification (1.6 mm)	Flame Retardant
PBT HB	HB	–
PBT V-2	V-2	Aluminium diethylenphoshinate
PBT V-1	V-1	Aluminium diethylenphoshinate
PBT V-0	V-0	Organic phosphinate
PP HB	HB	–
PP V-2	V-2	Radical generator
PP-EP HB	HB	–
PP-EP V-2	V-2	Tetrabromobisphenol A bis(2,3-dibromopropyl ether)
PA 6 HB	HB	–
PA 6 V-2	V-2	Melamine cyanurate
PA 6 V-0	V-0	Melamine cyanurate

From polymer pellets, polymer sheets 100 mm × 100 mm × 3 mm in size were prepared by a melt pressing process (pressure of 20 bar for 20 min, 30 min cooling time). PBT was dried for 4 h at 120 °C and immediately pressed at a temperature of 250 °C. To minimise moisture content, PA 6 was dried in a vacuum oven at 80 °C. The pressing temperature used for all PA 6 materials was 240 °C. PP was pressed at a temperature of 190 °C. After drying overnight, PP-EP specimens were injection-moulded using an industrial-scale injection moulding machine K 65/180/55 CX V by Krauss Maffei (Munich, Germany, *T*_max_ 210 °C, *T*_mould_ 30 °C). The polymer sheets were then cut into specimens of 100 mm × 6 mm × 3 mm.

### 3.2. Characterisation Methods

The thermal and thermo-oxidative stability of the materials was investigated by TGA under 30 mL·min^−1^ nitrogen flow or 30 mL·min^−1^ synthetic air flow, respectively. Then, 5 ± 0.1 mg samples (PBT) and 10 ± 0.1 mg samples for PP and PA 6 were heated from 30–900 °C in a Netzsch-TG 209 ASC F1 Iris (Netzsch, Selb, Germany) at a heating rate of 10 °C·min^−1^. For the beginning of decomposition, the temperature at 5 wt % mass loss (*T*_5 wt %_) was used. DSC was carried out using a Netzsch DSC 204F1 (Netzsch, Selb, Germany) under nitrogen atmosphere (50 mL·min^−1^). The samples (approximately 8.0 ± 0.1 mg) were heated from −40–250 °C at a heating rate of 10 °C·min^−1^ for three successive runs (heating, cooling and heating).

**Figure 13:**
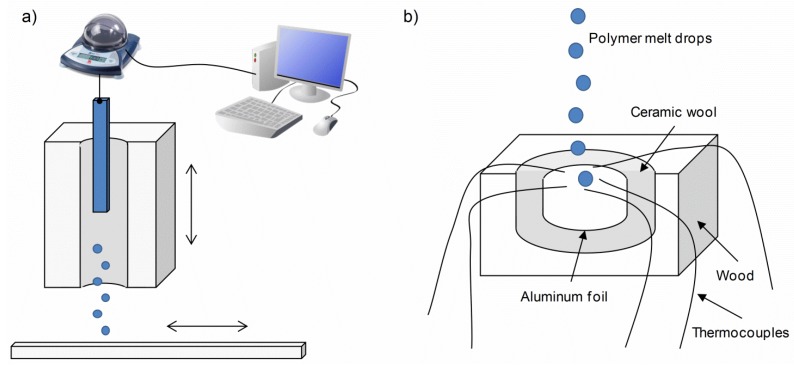
Scheme of the experimental setup for: (**a**) mass loss measurement; and (**b**) temperature measurement.

The details of the melt dripping experimental setup are given elsewhere [12,13], which, as shown in Figure 13a, consist of an 800-W movable electric furnace with a bore of 25 mm in diameter and 120 mm in length. The temperature of the calibrated furnace (air temperature in the middle of the furnace) is manually adjustable and limited up to 900 °C. The sample was hung on the digital mass balance and the furnace raised via pulley arrangement until the sample bottom was in the centre of the furnace. The mass loss as a function of time was recorded, and the drops were collected on an aluminium-foil-coated conveyer belt. For the drop temperature measurement (Figure 13b), the drops were collected just beneath the furnace in an isolated, aluminium-foil-covered container equipped with five thermocouples. The decomposition temperatures were taken as a guide value for the furnace setting in the mass loss experiment. The details are given elsewhere [13].

Rheological analyses of polymers and their molten drops were conducted using a plate-plate rheometer (Rheometer MCR 501, Anton Paar, Ostfildern, Germany) in oscillation mode at 230 °C for PBT and PA 6 and 190 °C for PP, respectively. The angular frequency was 100–0.1 rad·s^−1^ with a deformation amplitude of 0.5%.

## 4. Conclusions

The melt dripping behaviour of different polymers and the effect of flame retardants has been investigated. The work was carried out using three commercial polymers (PBT, PP, PP-EP and PA 6) with conventional flame retardants. The melt flow and dripping behaviour were highly dependent on the polymer, flame retardant, flame retardancy mode of action and additive.

The decreased total mass loss and higher drop temperature of the PBT V-2, PBT V-1 and PBT V-0 investigated are due to induced yield points at low shear rates. PTFE was used as an anti-dripping agent, crucially reducing melt flow and dripping. PP and impact-modified PP-EP featured similar dripping behaviours. Extensive decomposition resulted in extremely low viscosities due to the loss of the polymeric chain structure at higher furnace temperatures. The decomposition is enhanced by the flame retardants used, particularly at the beginning of decomposition, to achieve V-2 classification in PP V2 and PP-EP V2. PA 6 V-2 and PA 6 V-0 exposed to an external heating exhibited drops with decreased complex viscosity. The promotion of dripping of flame-retarded PA 6 by melamine cyanurate was apparent by a greater total mass loss, an earlier starting time of the mass loss and higher drop temperatures.

## Supplementary Materials

Supplementary materials can be accessed at: http://www.mdpi.com/1996-1944/8/9/5621/s1.
