# Supplementary material for: Influence of Flame Retardants on the Melt Dripping Behaviour of Thermoplastic Polymers
Source: Materials (Basel). 2015 Aug 27;8(9):5621–46. doi: 10.3390/ma8095267 (PMC5512619; doi:10.3390/ma8095267)
Supplement: Supplementary file 1 [file materials-08-05267-s001.pdf]

Supplementary Materials

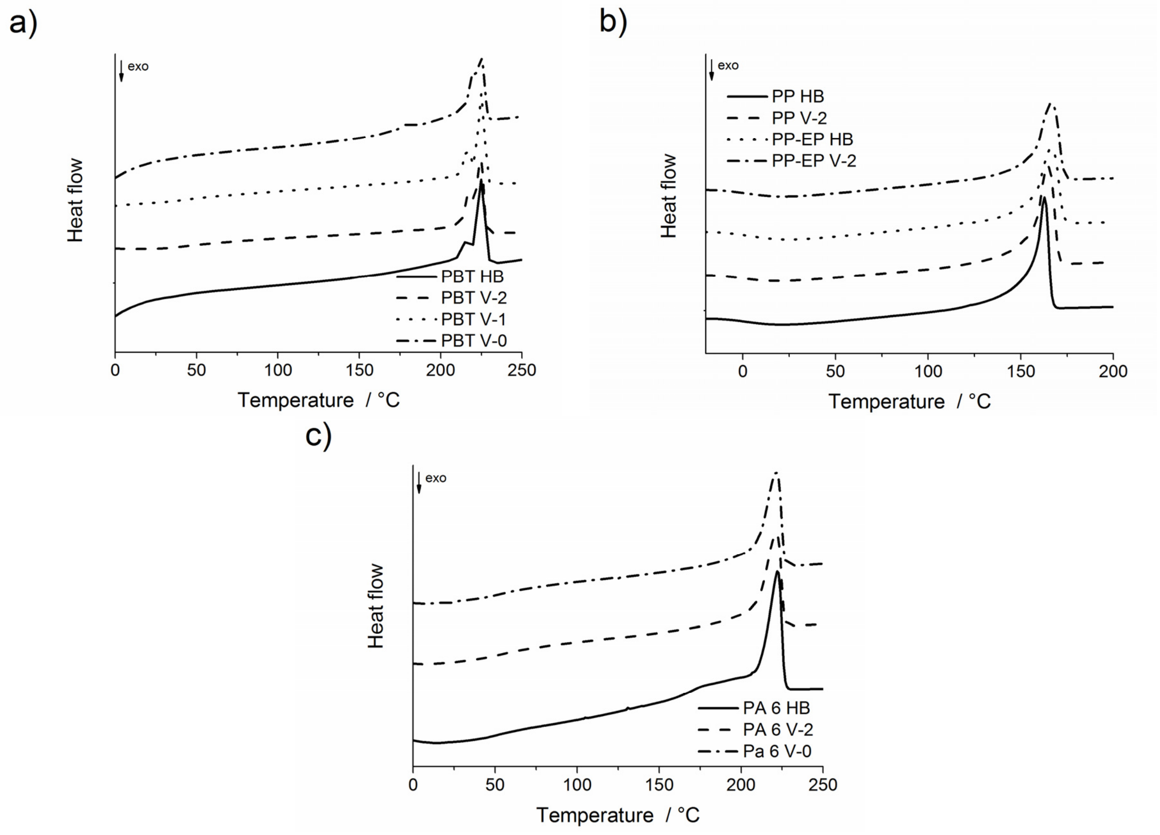

Figure S1. DSC under nitrogen: mass of (**a**) PBT HB, PBT V-2, PBT V-1, PBT V-0; (**b**) PP HB, PP V-2, PP-EP HB, PP-EP V-2; and (**c**) PA 6 HB, PA 6 V-2, and PA 6 V-0. Heating rate 10 °C min^{-1}.
